# COPLA, a taxonomic classifier of plasmids

**DOI:** 10.1186/s12859-021-04299-x

**Published:** 2021-07-31

**Authors:** Santiago Redondo-Salvo, Roger Bartomeus-Peñalver, Luis Vielva, Kaitlin A. Tagg, Hattie E. Webb, Raúl Fernández-López, Fernando de la Cruz

**Affiliations:** 1 Instituto de Biomedicina y Biotecnología de Cantabria (IBBTEC), Universidad de Cantabria-CSIC, Santander, Spain; 2 Departamento de Ingeniería de las Comunicaciones, Universidad de Cantabria, Santander, Spain; 3 Centers for Disease Control and Prevention, Atlanta, USA; 4 WDS, Inc., Atlanta, USA

**Keywords:** Horizontal gene transfer, Plasmid, Antibiotic resistance genes, Average nucleotide identity, Plasmid epidemiology

## Abstract

**Background:**

Plasmids are mobile genetic elements, key in the dissemination of antibiotic resistance, virulence determinants and other adaptive traits in bacteria. Obtaining a robust method for plasmid classification is necessary to better understand the genetics and epidemiology of many pathogens. Until now, plasmid classification systems focused on specific traits, which limited their precision and universality. The definition of plasmid taxonomic units (PTUs), based on average nucleotide identity metrics, allows the generation of a universal plasmid classification scheme, applicable to all bacterial taxa. Here we present COPLA, a software able to assign plasmids to known and novel PTUs, based on their genomic sequence.

**Results:**

We implemented an automated pipeline able to assign a given plasmid DNA sequence to its cognate PTU, and assessed its performance using a sample of 1000 unclassified plasmids. Overall, 41% of the samples could be assigned to a previously defined PTU, a number that reached 63% in well-known taxa such as the *Enterobacterales* order. The remaining plasmids represent novel PTUs, indicating that a large fraction of plasmid backbones is still uncharacterized.

**Conclusions:**

COPLA is a bioinformatic tool for universal, species-independent, plasmid classification. Offered both as an automatable pipeline and an open web service, COPLA will help bacterial geneticists and clinical microbiologists to quickly classify plasmids.

**Supplementary Information:**

The online version contains supplementary material available at 10.1186/s12859-021-04299-x.

## Background

Plasmids are essential elements in the dissemination of genes such as antibiotic resistance determinants among bacterial populations. The short-range evolution of bacterial genomes (as in epidemiological outbreaks) occurs more often by acquisition of mobile genetic elements carrying, for example, resistance determinants, than by point mutations creating new alleles with a selective advantage [1]. For this reason, obtaining a reliable method for plasmid analysis and classification is key for clinical microbiology. Several methods based on conjugative [2, 3] and replicative genes [4] for plasmid classification have been proposed. However, none of these methods is of universal application, since there is no single protein common to all plasmids. Recently, we reported a procedure to overcome these limitations, enabling the universal taxonomic classification of plasmids [5]. Using total average nucleotide identity (ANI) we showed that plasmids form defined PTUs (plasmid taxonomic units), genetically equivalent to plasmid species. These PTUs have characteristic host ranges and often harbor specific genetic determinants, such as virulence factors or antibiotic resistance genes. This opens the possibility of better tracking the propagation of plasmids across bacterial populations through genomic sequencing. Here we provide an algorithm for the automatic assignation of plasmids to PTUs, based on genomic sequence, which will help in the definition of the plasmid species responsible for outbreaks of antibiotic resistant strains [6].

## Implementation

### Building the PTU reference catalog

Our approach is based on a two-step strategy. First, a PTU reference catalog based on RefSeq84 plasmid sequences is built. By comparing a query sequence to this reference catalog, COPLA does assign its cognate PTU. In order to generate the reference catalog, an ANI network is constructed from the pairwise comparison of all curated RefSeq84 plasmids (NCBI, dataset from September 2017), as detailed in [5]. To build this network, plasmid genomes are used as nodes, and their pairwise ANI is calculated. Any pair of nodes is linked with an edge whenever they show an ANI score higher than 70% along 50% of the length of the smallest plasmid in the comparison. Clusters within the network are then identified using Hierarchical Stochastic Block Modeling (HSBM), which allows the inference of the statistical significance of the graph topological information [7]. HSBM has an inherent limitation when it finds a cluster much larger than the median size. It displays an inherent tendency to split clusters into average-size clusters, especially in networks with highly variability in cluster sizes. This subroutine has no biological logic, at least for plasmid clustering, so it needs to be tuned down, as we already discussed in [5]. COPLA follows the same strategy, so that PTUs are finally defined by merging HSBM clusters that fulfill the following criteria:

1. *Intercluster density:* two HSBM clusters (*C* and *D*) are merged if the number of inter-cluster edges (*m*_*C*,*D*_) is more than 50% of the maximum number of edges between both clusters, adjusted for the intra-cluster relative density. In mathematical terms:
  $$m_{C,D} > \frac{1}{2}n_{C} n_{D} \delta_{C}^{int} \delta_{D}^{int}$$
  being *n* and δ^int^ the number of vertices and intra-cluster edge density of the respective cluster.
2. *Size compatibility:* two HSBM clusters are merged if the median size of the plasmids of the cluster with smaller median size constituents (*C*) is larger than 50% of the median size of the larger cluster (*D*):
  $$\tilde{s}_{C} > \frac{1}{2}\tilde{s}_{D}$$

Thus defined, we identified 380 PTUs among RefSeq84 plasmids. A complete list of the plasmids used to build the reference network and their assignation to PTUs is found in Additional file 1. The reference network will be periodically updated, and a warning will be shown in the COPLA webpage.

### PTU prediction algorithm (COPLA)

Once the reference network and the PTU catalog were built, we implemented an algorithm to systematically assign any query plasmid sequence to its cognate PTU. The query plasmid can be used as input to the algorithm either as a complete genome, or as a set of plasmid contigs. Pairwise ANI scores between the query and each node in the reference network are calculated as in (2). The query node is then introduced in the network, linked to other nodes by edges when the ANI criterion is fulfilled. The query node is assigned a PTU by re-running the HSBM algorithm. However, this procedure is computationally intensive. To reduce the computation time, COPLA takes advantage of the bootstrap procedure used by the HSBM algorithm to refine the original partition. Instead of starting from an ab initio assignation, COPLA safely assumes that the addition of a single plasmid node to the 10,000 plasmid network is unlikely to alter the original partition. That is, the introduction of a new plasmid will not change the PTU assignation for the rest of plasmids in the network. Thus, the COPLA algorithm reuses the original partition, and performs an iterative reshuffling of the plasmids in the previously defined blocks, using a Monte Carlo algorithm. The query plasmid is included in the reshuffling. In order to identify the most likely allocation of plasmids in the partition, the algorithm proceeds iteratively by minimizing the Minimum Description Length (MDL) of the graph [7]. Since introduction of a new query should not, by the above definition, change the PTU assignation for the rest of the plasmids, the most likely allocation for the query is that which minimizes the MDL. Once this minimum has been reached, clusters are turned into PTUs by applying the size and inter-cluster criteria previously defined. A majority voting procedure is then used to label PTUs according to the reference tags carried by their members. The score in the assignation of a PTU to the query is based on the partition overlap [8] between all database plasmids with an annotated PTU that belong to the query cluster. The score indicates how much the clustering has changed due to inclusion of the query. COPLA also retrieves additional useful information, such as the MOB, MPF and Rep types of the query plasmid. This is achieved by retrieving the CDS from the sequence annotation, or detecting them using Prodigal, if absent [9]. CDS are then searched for MOB, MPF and Rep types using MOBSscan [2], CONJscan [3] and PlasmidFinder [4], respectively. This allows the user to check that the query has a typing scheme compatible with other members in the same PTU. Antimicrobial resistance (AMR) genes of the query plasmid are also identified with a blastn search (> 80% identity, < 1e−20 e-value) against the CARD database [10].

## Results

COPLA performance was benchmarked by carrying out two complementary validation tests. First, to assess COPLA accuracy, we randomly removed 1000 plasmids from the 9894-plasmid set in the curated reference database (RefSeq84). COPLA was run using each of these plasmids as query. A summary of the results is shown in Table 1, while the individual output and scores are shown in Additional File 2. As can be observed, COPLA correctly assigns 94% of the plasmids in the test set. The main source of error was in the assignation of plasmids that belong to PTUs with low intracluster density (due to the existence of subclusters, as occurs in PTUs such as PTU-F_E_ or PTU-F_K_), since the clustering algorithm is especially sensitive to variations in the constituent members of these clusters, as expected. Even with this caveat, COPLA only failed 6% of the times, so it can be considered as robust, accurate and consistent to the ground truth. Second, to assess the confidence of COPLA predictions, we randomly selected 1000 plasmids uploaded to NCBI after RefSeq84 was released (see Table 2) and run COPLA with each of these plasmids as query. The test set was obtained by downloading the RefSeq200 release (23,309 plasmid sequences), and removing those plasmid sequences already present in RefSeq84. A second filtering step was added, to eliminate sequences corresponding to genomic regions (NG_sequences in the NCBI notation) and incomplete sequences, which eliminated an additional 301 sequences. Additional file 3 lists the plasmids removed along with the rationale for their exclusion. After the filtering steps, a curated set of 12,561 new plasmids was obtained, from which 1000 were randomly chosen to evaluate COPLA.

**Table 1:** COPLA accuracy for a set of 1000 plasmids

Outcome	*Enterobacterales*	*Lactobacillales*	*Bacillales*	All samples
PTU correctly assigned	230 (89%)	88 (92%)	107 (94%)	935 (94%)
Cluster reconstructed^a^	4 (2%)	5 (5%)	3 (3%)	31 (3%)
Total correct predictions	234 (91%)	93 (97%)	110 (96%)	966 (97%)

1000 plasmids were randomly removed from the reference dataset (RefSeq84), consisting on 9894 plasmids. COPLA was run using each of these plasmids as query. The table indicates the number of cases (and percentage) for each prediction outcome for all samples and selected bacterial orders. The individual results can be found in Additional file 2

^a^As a result of the elimination of 1000 plasmids from the RefSeq84 sHSBM network, some PTUs containing 4–5 members fell below the threshold for PTU definition (at least four members). Thus, when a plasmid was assigned to one of these clusters, a PTU assignation did not follow (or resulted in a “new PTU assignation”), but in fact the result was correct, since the plasmid was assigned to the correct cluster

**Table 2:** Benchmark for 1000 new plasmids of RefSeq200 dataset for the most abundant bacterial orders

Outcome	*Enterobacterales*	*Lactobacillales*	*Bacillales*	All samples
PTU assigned				
Cases	259 (63%)	40 (46%)	25 (30%)	408 (41%)
Score	[0.98 ± 0.06]	[0.94 ± 0.11]	[0.96 ± 0.1]	[0.97 ± 0.08]
New PTU				
Cases	19 (5%)	6 (7%)	2 (2%)	41 (4%)
Score	[0.84 ± 0.22]	[0.89 ± 0.17]	[0.75 ± 0.35]	[0.83 ± 0.24]
Not assigned				
Cases	131 (32%)	41 (47%)	55 (67%)	551 (55%)
Score	[0.99 ± 0.05]	[1 ± 0.01]	[1 ± 0.0]	[1 ± 0.05]

Number of cases (and percentage) for each prediction outcome. Mean and standard deviation of the prediction scores for each outcome class are additionally provided (in square brackets). More detailed results in Additional file 4

Query plasmids yield one of three alternative outcomes when using the PTU prediction algorithm COPLA as explained above (see Additional file 4 for the individual results):

1. The query is assigned to an existing PTU. This happened in 408 cases, representing an overall 41% of positive assignation in the testing set. This figure was increased to 63% (259 out of 409 query plasmids) in the case of *Enterobacterales.* This increase in the identification rate was to be expected, since enterobacterial genomes have been more thoroughly sampled than other taxa. When there is a positive PTU assignation, COPLA retrieves the PTU of the query along with the score of the prediction.
2. A plasmid may cluster with less than 3 plasmids in the reference dataset, thus no PTU may be assigned. In this case COPLA indicates that no PTU assignation is possible within this reference network.
3. A plasmid sequence clusters into a group of 3 or more plasmids with no previously assigned PTU. This may happen because clusters are only named in case the resulting PTU has at least 4 member plasmids. The addition of the query may thus form a 4-member group which corresponds to a potentially new PTU. In the testing set, this happened on 41 occasions (4% of the times). In this case COPLA indicates that the plasmid is part of a new, still unnamed, PTU.

**Fig. 1:**
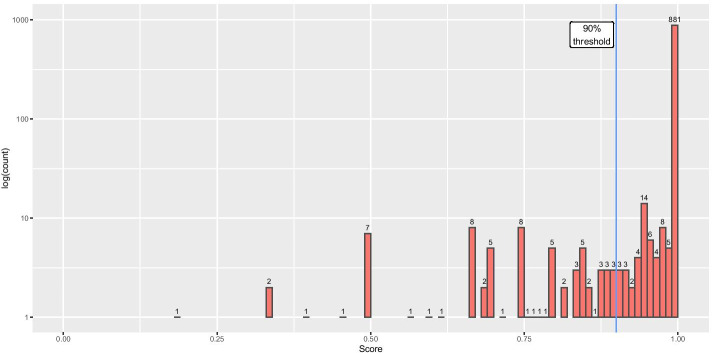
Score distribution for 1000 plasmids sampled from RefSeq200, not present in the COPLA reference database (RefSeq84). The figure displays a semilogarithmic plot of the number of plasmids resulting in each given score

According to the score output, ~ 88% of all queries in the testing set achieved prediction scores > 99% (see Fig. 1). Plasmids with lower scores may represent cointegrates between different PTUs or incorrect assemblages from NGS data, which is often a problem for plasmids [11]. Cointegrates can sometimes be identified by the observation of two different MOB classes in the same query, but this is not always the case. Furthermore, MOB typing is unavailable for non-mobilizable plasmids. For these reasons we recommend at least a 90% score in order to validate a PTU assignment. A 90% score indicates that for a 10 member PTU, the query has conflicting data for clustering 1 of the members. Adopting this 90% score threshold, COPLA confidently assigned 93% of the 1000 samples.

## Discussion

**Fig. 2:**
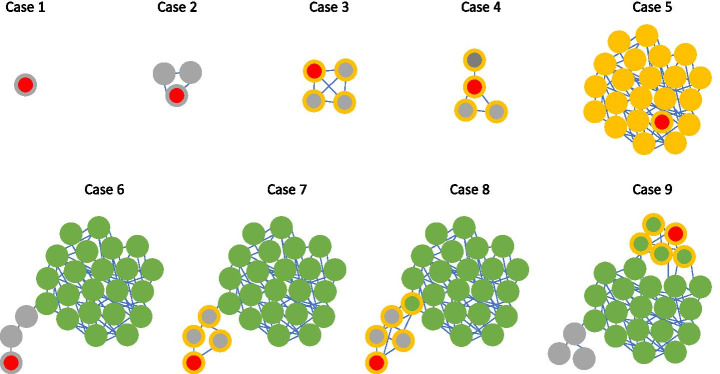
Representative prediction outcomes. The query plasmid is represented by the node with the red inner circle. For all other nodes, the color of the inner circle represents the PTU assigned in the reference database (i.e. using only RefSeq84 plasmids). The outer ring colors represent the PTU assigned by COPLA. Yellow represents the PTU assigned to the query, green corresponds to nodes belonging to a different PTU, and grey represents not assigned PTUs. Case 1: the query represents a singleton. Case 2: the query belongs to a cluster with one or two members. A PTU cannot be assigned. Case 3: the query belongs to a cluster with three members. COPLA predicts a “new putative PTU”. Case 4: the query links together isolated plasmids to organize a 4-member cluster. COPLA predicts a “new putative PTU”. Case 5: the query clusters with the members of a known PTU. COPLA predicts that query belongs to that PTU. Case 6: the query links peripherally to a cluster corresponding to a known PTU. However, either the number of connections is not enough to fulfill the intercluster density rule, or the size of the query is not compatible to that of the PTU (see “Building the PTU reference catalog” in Implementation). COPLA output indicates that no PTU can be assigned to the query. Case 7: the query links peripherally to a cluster corresponding to a known PTU. The query organizes a subcluster of four members that does not fulfill the rules to integrate in the PTU. COPLA output predicts a “new putative PTU”. Case 8: As in case 7, the query organizes a subcluster that does not fulfill the rules to integrate in the PTU. Furthermore, it drags one member of the PTU to the new cluster. COPLA output predicts a “new putative PTU”. Case 9: the query significantly alters the structure of a known PTU. COPLA output predicts a “new putative PTU”. It also warns that “query is related to PTU-… plasmids”. See additional details and explanations in the main text (Discussion)

Results yielded by COPLA can be better understood by looking at the alternative outcomes for a given query, which are shown in Fig. 2. The first outcome, that is, the assignation of the query to a PTU already present in the network is trivial, and it is shown in case 5 in the Figure. The second outcome, in which no PTU can be assigned to the query, is shown in cases 1, 2 and 6 in Fig. 2. In case 1, the query represents a singleton (a unique plasmid architecture never sampled before in the reference set). In case 2, the query links to a cluster with less than 3 other plasmids. Cases 1 and 2 indicate that the query represents a member of a new PTU, but the number of genomes ascribed to it is still too low to name the PTU with statistical significance. Case 6 occurs when a plasmid is linked either directly or indirectly to a known PTU, but the number of connections is either not enough to fulfill the intercluster density, or the size of the plasmids are not uniform. This may occur for plasmids carrying integrons, transposons or other mobile genetic elements whose size is large, compared to the size of the given plasmid. Plasmid cointegrates (either real cointegrates or artifacts of sequence assembly) may also yield a similar result. In all these cases, COPLA output indicates that no PTU can be assigned to the query.

The third outcome, that is, the assignation of the query to a PTU that was previously undefined, may occur in a number of ways, as shown in cases 3, 4, 7, 8 and 9 in Fig. 2. The query may be assigned to a cluster with at least 3 other members that did not have a PTU assignation in the reference network (cases 3 and 4). In these cases, the COPLA output indicates that a new potential PTU has been identified for the query. The query may also cluster with > 4 plasmids which showed links to existing PTUs, but there was no PTU assignment for all (case 7) or most of them (case 8). These cases, specially case 8, must be examined carefully. Often, this kind of result is produced by the clustering artifacts produced by large mobile genetic elements or when the query is in fact a cointegrate of two different plasmids. In these cases, it may help to examine the additional information of the COPLA output, such as the MOB, MPF and Rep types. If present, they should coincide with those of the proposed members of the new PTU.

The most complicated case is 9, in which the addition of a new query “breaks” an existing PTU, yielding a new statistically significant group. In our experience with the 1000 plasmids tested, this only happened in the case of PTU-F_E_, a PTU that includes *Escherichia coli* plasmid F. As reported in our previous analysis, PTU-F_E_ is controversial [5]. It shows a borderline intercluster density, and various “nascent” subgroups may be identified within it [5]. Thus, any query plasmid that forms a new PTU with members of PTU-F_E_ must be analyzed with caution. The most conservative assumption is to assume that the query belongs to the larger PTU (PTU-F_E_, most likely) but it would be advisable to conduct further genomic comparisons to determine if the partition identified by COPLA is significant, and a sub-group with a coherent genome can be identified. This latter situation may represent a group of plasmids in the process of speciation, where increasingly lower recombination causes the progressive separation of the different subspecies in the graph [5].

In all cases, the identification of a new PTU should be followed by further genomic inspection and characterization. COPLA is optimized for the assignation of a query to a PTU, which is the most frequent scenario for researchers coming with a new genomic sequence which may contain untyped plasmids. The robust definition of new PTUs, however, is better achieved by the simultaneous addition of many new sequences, and the ab initio execution of the HSBM procedure, which may take several days of computation, and much longer time for bioinformatic analysis. For this reason, COPLA may yield a query plasmid as untyped when working with a reference network, but type it robustly when a larger reference set is used. Currently, almost 60% of the query plasmids cannot be typed because they lack a sufficient number of similar genomes in the reference set to constitute a PTU. This indicates that the number of bacterial plasmids identified in the databases is still a small fraction of the true PTU diversity. However, with the enrichment of the databases in each new RefSeq release, the predictive power of COPLA is expected to increase. This will be achieved by the regular renewal of the reference database, which will be referred to the RefSeq release it corresponds to.

## Conclusions

COPLA is a tool aimed for rigorous plasmid classification, based on the concept of PTU. It will be useful for non-specialists with an interest in various aspects of plasmid biology. Using a score threshold > 90%, COPLA does confidently assess > 93% of plasmids not contained in the reference database. For plasmids of the *Enterobacterales* order, COPLA reaches a rate of 63% positive plasmid assignation to currently defined PTUs, while for the whole bacterial domain, it reaches 41%. To facilitate its widespread use, COPLA was deployed as a free access web service, providing the user not only with the PTU assignation of the query plasmid, but also its potential host-range, related taxonomic information such as the MOB class, potential family (MPF type), and predicted antibiotic resistance genes.

## Supplementary Information

**Additional file 1.** List of reference plasmids. A complete list of the plasmids from release 84 of the NCBI RefSeq Plasmid database used to build the reference network and its assignation to PTUs, the current COPLA reference database.

**Additional file 2.** Accuracy of COPLA algorithm for 1000 plasmids randomly removed from the reference dataset (RefSeq84). The columns of the Excel file show ground truth, predicted PTU assignment, associated host range, and prediction score for each of the 1000 query plasmids.

**Additional file 3.** RefSeq200 plasmid dataset. A complete list of the plasmids from release 200 of the NCBI RefSeq Plasmid database. This dataset is the source of the plasmids used to evaluate COPLA performance.

**Additional file 4.** Benchmark for 1000 new plasmids of RefSeq200 dataset. Predicted PTU, host range and prediction score of COPLA for randomly chosen 1000 plasmids not present in the current COPLA reference database.

## Data Availability

Project name: COPLA. Project home page: Web service: https://castillo.dicom.unican.es/copla; public code repository: https://github.com/santirdnd/COPLA. Operating system(s): UNIX, web service. Programming language: Python, Bash, Perl. Other requirements: BLAST+2.9.0 or higher, Prodigal v2.6.3 or higher, HMMER v3.3 or higher, PlasmidFinder 2.1 or higher, MacSyFinder 1.0.5 or higher, graph-tool 2.33 or higher, GNU Parallel 20161222 or higher, ani.rb (from https://github.com/lmrodriguezr/enveomics). License: GPLv3. Any restrictions to use by non-academics: None. The datasets analyzed during the current study were obtained from NCBI RefSeq Plasmid database, releases 84 and 200 (ftp://ftp.ncbi.nlm.nih.gov/refseq/release/plasmid/). The accession numbers of the plasmids contained in both releases are listed in the Additional files 1 and 3. The databases used during the current study are available in the COPLA databases repository, https://castillo.dicom.unican.es/zaguan/Copla/Copla_databases_RS84.tar. These databases were derived from the following public domain resources: The MOBscan relaxase database (https://castillo.dicom.unican.es/mobscan_about); the CONJscan MPF typing database (https://github.com/gem-pasteur/Macsyfinder_models, downloaded on 2019-05-30); the Comprehensive Antibiotic Resistance Database (CARD) database version 3.1.0 (https://card.mcmaster.ca/download, downloaded on 2020-10-15); the PlasmidFinder replicon database (https://bitbucket.org/genomicepidemiology/plasmidfinder_db.git, downloaded on 2019-07-31).

## Abbreviations

PTU: Plasmid taxonomic unit
DNA: Deoxyribonucleic acid
ANI: Average nucleotide identity
HSBM: Hierarchical stochastic block modeling
CDS: CoDing sequence
MPF: Mating pair formation
AMR: Antimicrobial resistance genes
MDL: Minimum description length
ORF: Open reading frame
